# Low lateral right atrial scar: implications for the diagnosis of cavotricuspid isthmus block following ablation for atrial flutter

**DOI:** 10.1007/s10840-025-02204-7

**Published:** 2026-01-16

**Authors:** Oholi Tovia-Brodie, Raul Mitrani, Alex Velasquez, Litsa Lambrakos, Jeffrey J. Goldberger

**Affiliations:** 1 Division of Cardiology, Jesselson Integrated Heart Center, The Eisenberg R&D Authority, Shaare Zedek Medical Center, The Hebrew University of Jerusalem, Jerusalem, Israel; 2 Division of Cardiology, University of Miami, 1120 NW 14th St., Suite 1123, Miami, 33136 FL USA

**Keywords:** Atrial flutter, Mapping, Catheter ablation, Cavotricuspid isthmus

## Abstract

**Introduction:**

High density 3D mapping for typical atrial flutter (AFL) ablation can provide a highly detailed voltage and activation map. Post-ablation mapping is typically used to identify cavotricuspid isthmus (CTI). Low voltage areas in the right atrium may yield misleading mapping results.

**Methods and results:**

3D electroanatomic voltage and activation mapping of the right atrium (RA) was performed in 15 patients with typical AFL, using 3D high density mapping with a Halo or Pentaray catheter pre- and post-ablation. Activation and entrainment mapping confirmed CTI dependent AFL in all patients. Mean number of map points was 611 ± 312. Lateral low voltage areas were seen in 11 (73%) patients. Post-ablation activation map during CS pacing (*n* = 8) demonstrated latest activation on the lateral wall aligned with the low voltage areas (in 6 of 8 patients), and in 3 (27.3%) patients this was later than just lateral to the CTI ablation line, masquerading as a gap in the ablation line. However, bidirectional block was confirmed by differential pacing, widely split double potentials on the ablation line and non-inducibility. Failure to recognize this misleading activation map in 2 patients resulted in delivery of significantly more ablation lesions (34 vs. 19, *p* = 0.0007).

**Conclusion:**

Areas of low voltage in the low lateral right atrium may lead to slow conduction and delayed activation in this area, in some cases even greater delay than just lateral to the CTI ablation line mimicking a gap in the ablation line. Comparing pre-ablation voltage to post-ablation activation map can identify areas of low voltage with slow conduction. The use of other maneuvers can prove bidirectional block and avoid further unnecessary RF delivery.

**Graphical Abstract:**

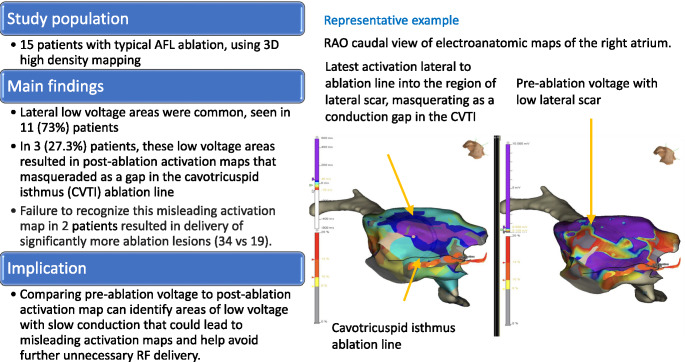

## Introduction

Typical atrial flutter (AFL) represents the most common atrial macro-reentrant tachyarrhythmia. Catheter ablation is the most effective therapy to maintain sinus rhythm. It is clearly superior to amiodarone [1, 2], and has a high success rate. It is therefore recommended as first line therapy for symptomatic typical AFL [3]. While historically catheter ablation of typical cavotricuspid isthmus (CTI) dependent AFL was performed using fluoroscopic guidance only, currently three dimensional (3D) electroanatomic mapping is commonly used in ablations of atrial arrhythmias, with proven benefits in the mapping and ablation of right sided scar related or left sided atypical flutter [4–8]. For CTI dependent AFL, the key steps for the procedure are establishing the diagnosis, performing the ablation, and confirming CTI conduction block. In this report, we describe a case series of patients in whom the presence of low lateral right atrial scarring created a post-ablation conduction pattern mimicking breakthrough conduction through the CTI ablation line.

## Methods

### Patient population

Based on initial observations of the impact of low right atrial scarring on the diagnosis of CTI block post-ablation in 2 patients, we evaluated consecutive patients who underwent first time ablation for typical CTI-dependent AFL between June 2017 and August 2018 and had pre-ablation 3D high density mapping with a multipole catheter.

## Ablation procedure

The patients were brought to the EP lab in the post absorptive state after informed consent was obtained. The groins were prepped and draped in the usual sterile fashion and femoral venous sheaths were placed in usual fashion through which electrode catheters were inserted and advanced to the heart. When using the EnSite (*St. Jude Medical*,* Inc.*,* St. Paul*,* MN*,* USA)* mapping system, electroanatomic mapping of the right atrium was performed with the HALO™ XP Tricuspid Mapping Catheter (JNJ Medtech), and a decapolar catheter (Inquiry™, SJM) placed in the coronary sinus. When using the CARTO^®^ (*Biosense Webster*,* Diamond Bar*,* USA)* system, electroanatomic mapping of the right atrium was performed with the PENTARAY™ NAV catheter. Activation mapping and entrainment mapping were used to confirm a CTI-dependent flutter. Normal electrogram voltage was defined as > 0.5mV and scar < 0.1mV. Two electrophysiologists assessed the scar size qualitatively as small or medium. Using a *standard 8-mm tip or an irrigated ablation catheter* through a long sheath, RF energy was delivered from the tricuspid valve to the IVC, until a bidirectional line of block was achieved. After a waiting period of 30 min, we reconfirmed bidirectional line of block with 3D electroanatomic mapping (transisthmus conduction time) and differential pacing. Complete post-ablation maps of the right atrium were not routinely performed, particularly when bidirectional block was evident by other means. Post-procedural follow-up was done at electrophysiology office visits.

### Statistical analysis

Continuous variables are expressed as mean ± standard deviation, if normally distributed. Categorical variables are expressed as frequency (percentage) and tested with the Fisher’s exact test. Means are compared using Student’s t test or the Mann– Whitney U test. Statistical analyses were performed with SPSS for Windows (release 17.0, SPSS Inc., Chicago, IL, USA).

## Results

**Fig. 1:**
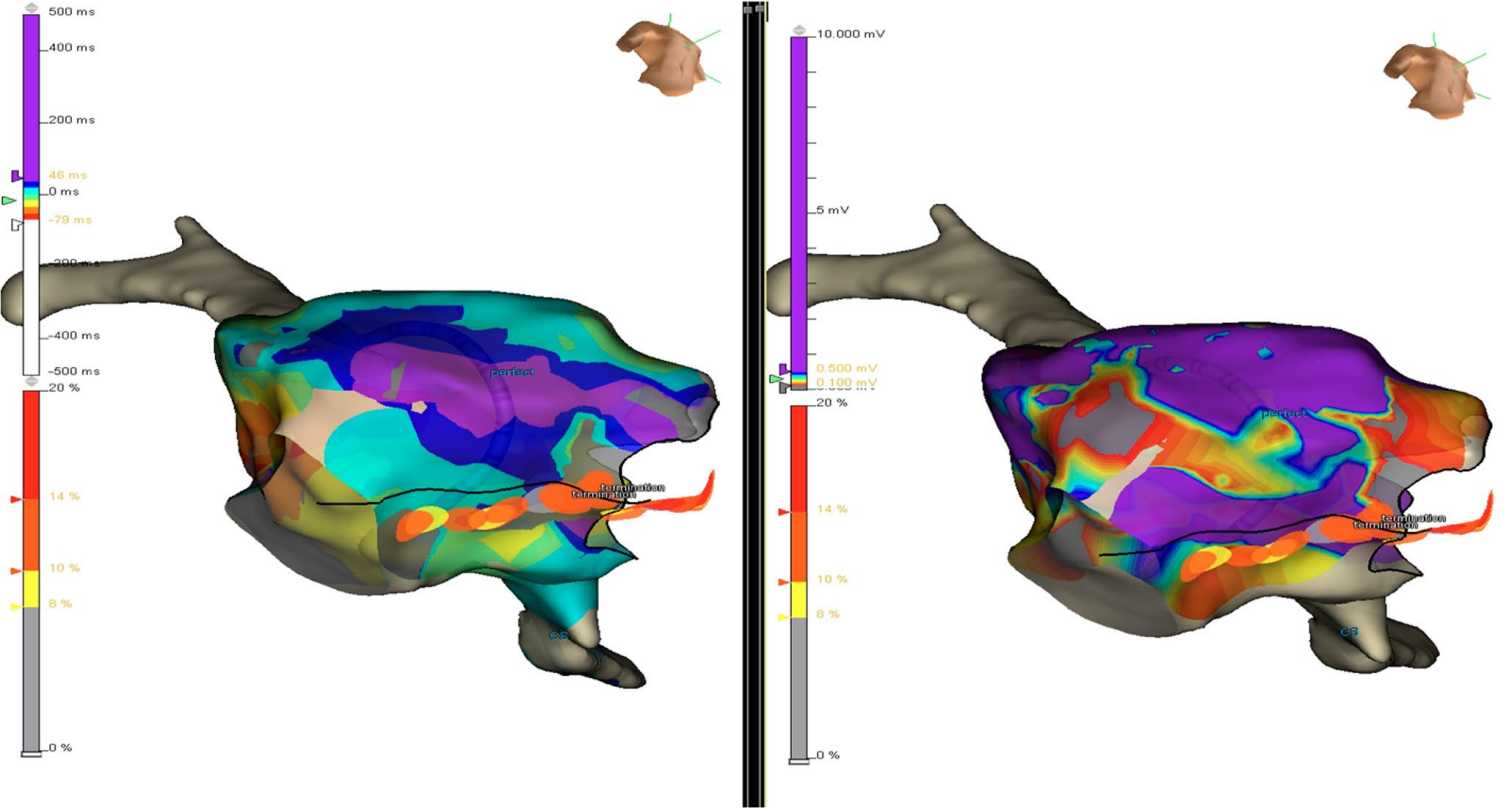
RAO caudal view of electroanatomic maps of the right atrium. **A**: post ablation activation map during coronary sinus pacing, demonstrating latest activation on the lateral wall, not adjacent to the ablation line (marked in black), masquerading as a gap in the ablation line. **B**: pre-ablation voltage map demonstrating low voltage areas aligned with the latest activation noted on the lateral wall in panel A

**Fig. 2:**
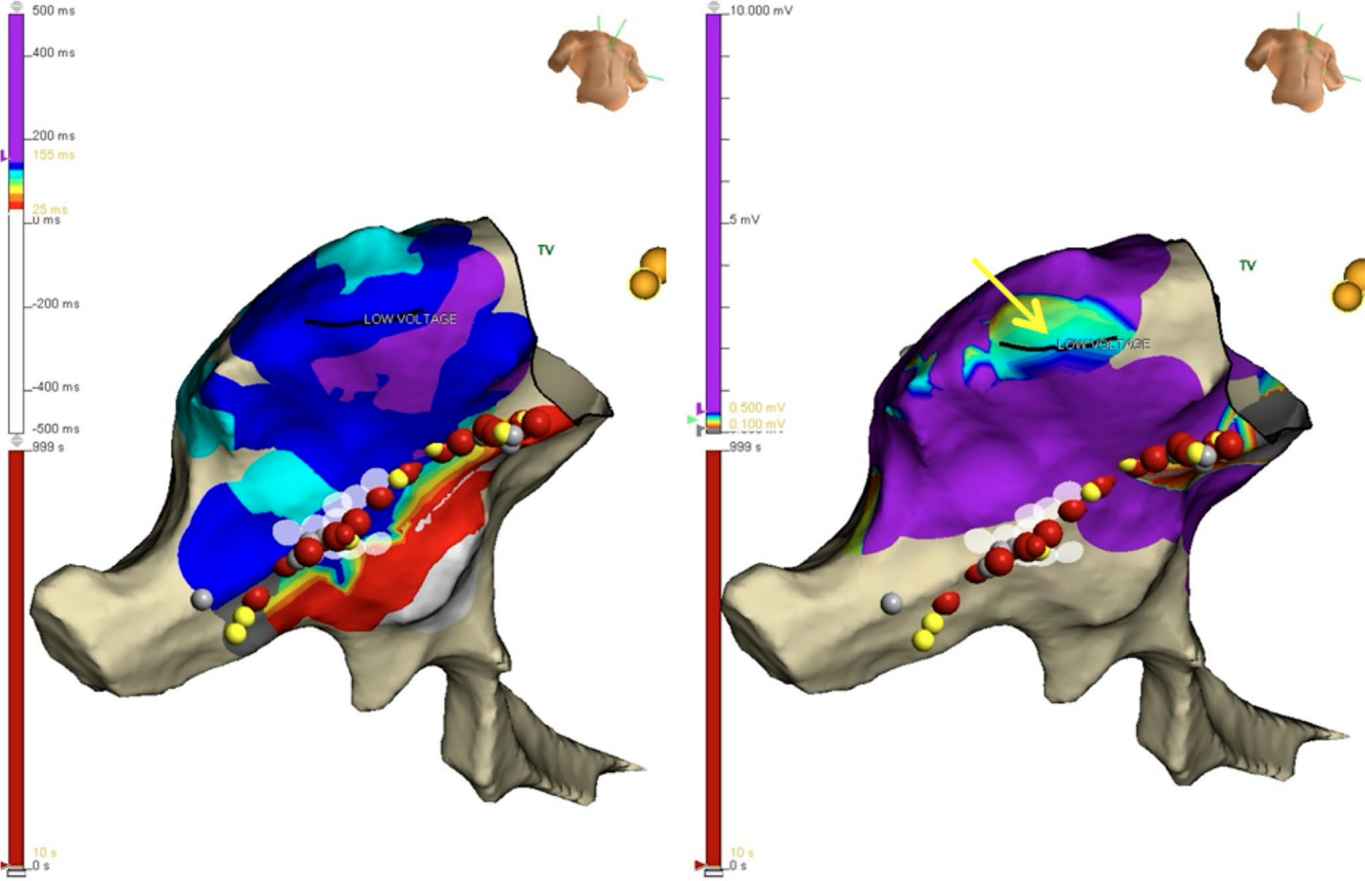
Inferior view of electroanatomic maps of the right atrium. **A**: post ablation activation map during coronary sinus pacing, demonstrating latest activation on the lateral wall, not adjacent to the ablation line (red and yellow dots), masquerading as a gap in the ablation line. **B**: pre-ablation voltage map demonstrating low voltage areas aligned with the latest activation on the lateral wall (arrow)

**Fig. 3:**
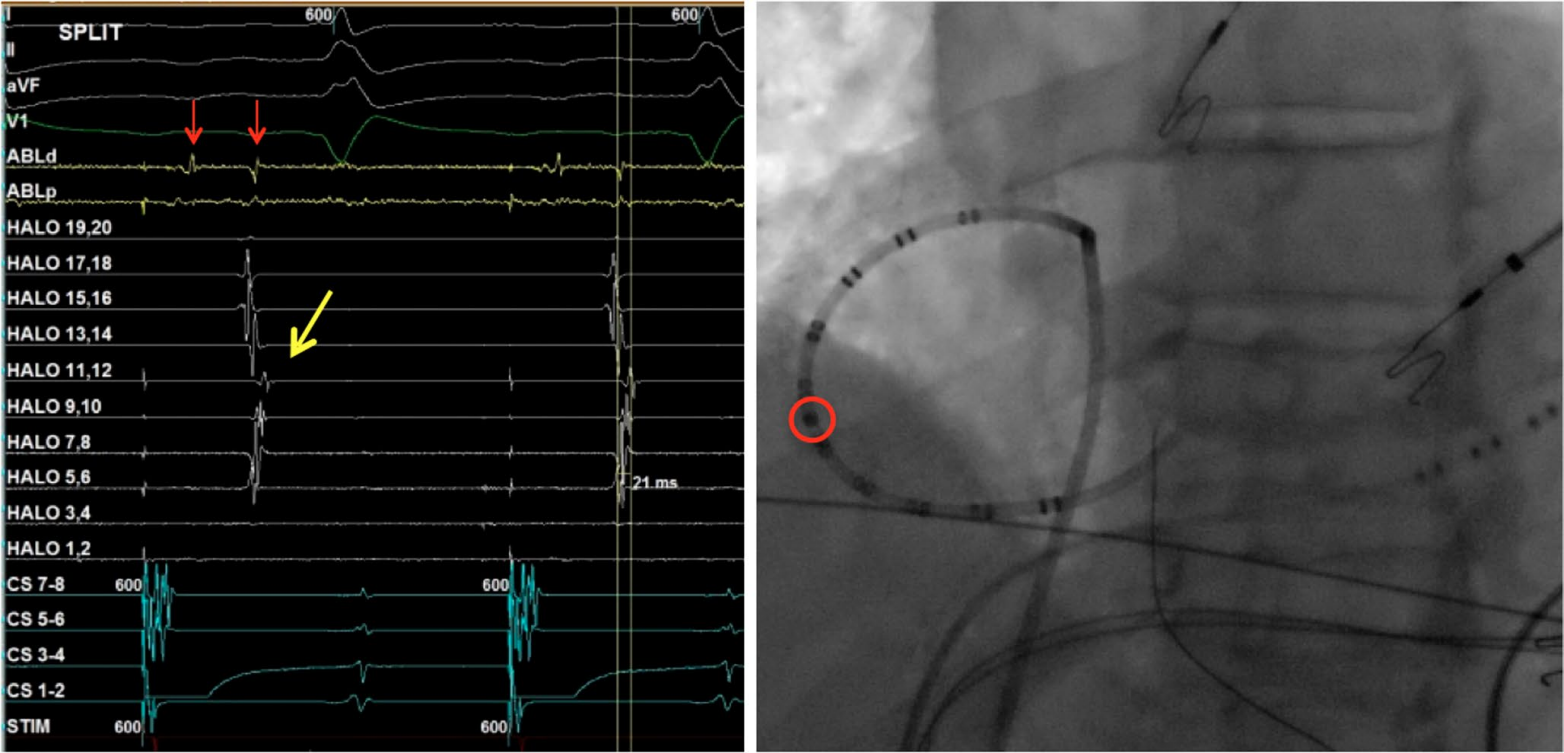
ECG and intracardiac recordings from the ablation catheter (ABLd, ABLp), the multielectrode Halo catheter, and the coronary sinus (CS) during CS pacing at cycle length 600 ms post-ablation with a LAO fluoroscopic image of the Halo catheter position. The latest activation is recorded on electrodes 11–12 (yellow arrow on electrogram and red circle on fluoroscopic image), positioned in the low right atrium with a 21ms delay compared to electrodes 5–6 on the Halo. Widely split potentials separated by 109 ms and with electrogram inversion are recorded on the ablation catheter (red arrows)

The study population consisted of 15 patients. Baseline patient characteristics are presented in Table 1. Twelve (80%) patients were male. The mean age was 62.8 ± 9.3 years. All patients presented with typical counterclockwise AFL, cycle length 243 ± 24 ms. Procedural characteristics are presented in Table 2. Activation and entrainment mapping confirmed CTI dependent AFL in all patients. Mean number of map points was 611 ± 312. Lateral low voltage areas were seen in 11 (73%) patients, of whom 5 were qualitatively small and 6 were qualitatively medium size. Post-ablation activation map during CS pacing (*n* = 8) demonstrated latest activation on the lateral wall aligned with the low voltage areas (in 6 of 8 patients). In particular, in 3 of these patients (2 medium and 1 small size scar) activation in the lateral right atrium was later than just lateral to the CTI ablation line, masquerading as a conduction gap in the ablation line (examples in Figs. 1 and 2). Two of these 3 patients had prior cardiac surgery (1 aortic valve repair, 1 sinus venosus atrial septal defect repair). Figure 3 provides electrograms recorded from the Halo post-ablation during CS pacing in which the latest activation is seen in the low right atrium (electrodes 11–12 on the Halo) while the more distal electrodes closer to the CTI ablation are activated earlier, consistent with “apparent conduction” over the CTI. However, bidirectional block was confirmed by differential pacing, widely split double potentials on the ablation line (seen in Fig. 3), and non-inducibility. Failure to recognize this misleading activation map in the 2 index patients resulted in delivery of significantly more ablation lesions than was necessary to achieve presumed CTI conduction block (34 versus 19); neither patient had recurrent CTI dependent AFL. No complications occurred. Follow-up was available for 14/15. No clinical recurrences of typical AFL occurred at median follow-up of 64 months, but 8 patients developed atrial fibrillation or atypical atrial flutter.

**Table 1:** Patient characteristics

Characteristic	
Age (years)	62.8 ± 9.3
Sex	12 M/3F
Hypertension	10 (67%)
Diabetes mellitus	5 (33%)
Coronary artery disease	5 (33%)
Atrial fibrillation	4 (27%)
Antiarrhythmic drugs	2 (13%)
LVEF (%)	53.7 ± 11.6
Left atrial volume index	39.7 ± 16.1

 14(93)

**Table 2:** Procedural Characteristics

**Procedural Characteristics**	**n(%)/ mean±SD**
Baseline rhythm AFL	14 (93)
AFL CL (ms)	243 ± 24
Termination during ablation	12 (80)
Number of RF lesions	21 ± 7
Ensite mapping system	14 (93)
Number of map points	611 ± 312
Minimal voltage (mV)	0.03 ± 0.01
Maximal voltage (mV)	9.9 ± 3.2
Lateral scar	11 (73)
Post activation map	8 (53)
Post map identified latest activation aligned with the low voltage areas	6 (40)
Post map masquerading as a gap	3 (20)

## Discussion

In this report, two index cases identified post-CTI ablation activation patterns that were altered due to low voltage areas in the low right atrium. Specifically, due to delayed conduction into these low voltage areas, they were activated later than just lateral to the CTI ablation mimicking a pattern of conduction breakthrough through the CTI. Further detailed mapping in a cohort of patients with AFL identified that delayed conduction into low voltage areas commonly occurs and depending on the extent and location of these low voltage areas may occasionally create a post-ablation conduction pattern during CS pacing that mimics conduction breakthrough. It is important to recognize this pattern, specifically low voltage in the low right atrium, causing delayed conduction to this area, so that additional unnecessary ablation lesions are not delivered.

The clinical success rate for catheter ablation of AFL is ~ 85%^1^ Multiple studies over several decades demonstrate acutely successful achievement of bidirectional CTI conduction block in 89–100% of cases [2–10]. Of note, precise criteria for bidirectional CTI conduction block are typically unstated. Anatomic variants are likely the most common cause of failure to achieve bidirectional CTI conduction block [11]. We report an electrophysiologic explanation for apparent failure to achieve bidirectional CTI conduction that is incorrect due to delayed activation in low voltage (scar) areas in the low RA. Low voltage or scarred areas in the RA may have multiple etiologies, including post-surgical scarring and atrial myopathy [12]. Although this is likely an uncommon occurrence, it is important for the operator to recognize this possibility to mitigate further unnecessary ablation in an attempt to alter this activation sequence. The diagnosis of bidirectional CTI conduction in patients with this pattern of activation can be confirmed in a number of ways. As shown in Figs. 1 and 2 and a comparison of the post-ablation activation to the voltage map can establish the pathophysiologic basis for this phenomenon. Further confirmation with widely split electrograms along the length of the CTI ablation line would be confirmatory. Vector activation on both sides of the line can also be confirmatory. Other etiologies of “pseudoconduction” across the CTI, such as transcristal conduction, have been reported [13]. 

In the present report, the voltage maps were acquired prior to ablation. However, as the regions of low voltage are remote from the ablation line, this could be done, as needed, post-ablation. This provides another potential advantage to the use of 3D-mapping systems to guide ablation of typical CTI-dependent AFL. They have been shown to reduce total x-ray exposure compared to the fluoroscopy-guided approach [9–11], and aid in the anatomic reconstruction of CTI, which may help guide ablation in patients with complex CTI anatomy [12]. Activation mapping during CTI-dependent AFL can help identify the AFL to be CTI-dependent and identify other reentrant circuits such as upper and lower loop reentry, while post-ablation mapping can be helpful in establishing CTI conduction block.

### Limitations

The limitation of the present study is that we cannot define the precise incidence of this phenomenon as the observation is centered around two index cases that we identified. As detailed mapping of the right atrium is not typically performed in patients with CTI-dependent AFL, we identified only a limited number of such patients which may represent a biased sample. Yet, we did identify an additional patient with the same pattern of activation as in the index cases. While we expect this pattern to be uncommon, it is important that this possibility be recognized as an explanation for an altered activation sequence post-CTI ablation.

## Conclusions

CTI ablation for treatment of CTI-dependent typical AFL is highly successful. Patients with typical AFL may have diverse underlying atrial substrate. We have identified an atrial substrate – delayed conduction in the low lateral right atrium – that can produce an activation pattern during coronary sinus pacing following CTI ablation that appears to show conduction breakthrough the CTI, but instead reflects slow conduction and later activation of this tissue compared to just lateral to the CTI ablation. Recognition of this potential confounding activation after CTI ablation is important to avoid further unnecessary ablation. Figure 1.
